# Supplementary material for: Free and Open Source Software organizations: A large-scale analysis of code, comments, and commits frequency
Source: PLoS One. 2021 Sep 23;16(9):e0257192. doi: 10.1371/journal.pone.0257192 (PMC8460050; doi:10.1371/journal.pone.0257192)
Supplement: Attachment — Submitted filename: Review PONE-D-20-37277R1.pdf [file pone.0257192.s002.pdf]

This article undertakes a longitudinal study of participation in FOSS projects, considering commit frequency, lines of code, and code comments. The work concludes that contributors are less active now, and that this decline is more strongly associated with large organizations than smaller ones. This finding is associated with three outcomes: the relationship between peer production and commercial production is maturing, the role of large organizations as intermediaries is declining, and the "likelihood of new big formal organizations" managing "emerging" projects is "systematically declining". The work concludes that this result suggests that open organizing is a "pipe dream".

This paper tackles an important concern with an ambitious scope. The writing is clear, and I appreciate the attention to a range of FOSS-related organizations. The work has strong potential to contribute to the field, with implications for computer science, social computing, and organizational studies.To the extent these organizations are in a state of failure, we need to know about it.

Overall, however, I find that this project faces a crossroads; there is a disconnection between the evidence developed and the argument made, and one side or the other needs to give way. In particular, there needs to be a clear connection between all of the core elements of the paper. To assist in tracking my argument in parallel with the paper's argument, I have broken my concerns into six themes below: the unit of analysis, the sample, the measures, the methods, the connection between the analysis and the discussion, and the ultimate conclusions. However, the gist of my concern (inconsistency between the design section, analysis section, and introduction/discussion/conclusions) is threaded throughout the paper, so some of my concerns as I describe them may seem to repeat/reprise previous concerns; for this I apologize in advance.

1. The unit of analysis

Multiple units of analysis are introduced: the individual contributor, the specific project, and the organization under whose umbrella the project was either born or has migrated to over time. The shifting unit of analysis is unfortunate because it means that many of the claims as stated are not sustained by the analysis provided.

For example, the paper concludes that contributors are less active now; however, to do so, the analysis would need to make within-person comparisons, tracking each unique contributors over time, and comparing their early work volumes to their later work volumes, however this analysis is not what was done and so a claim at the individual level is not supported. The claim could be rescoped to state that the organization is receiving fewer contributions, but since the contributions are made at the level of the project and the number of projects has varied through time, it seems expected that contribution volumes would do likewise.

Organizations are analyzed as an aggregate of their projects' productivity. However, several of the conclusions and discussion points concern organizational effectiveness at providing other kinds of services, i.e. being an intermediary, offering a community of practice, advocating for

open production in society as a whole. However, these other activities are not part of the data developed. Ultimately there needs to be more connection between the data and the way the results and claims are scoped.

2. The sample composition

Building from concerns about the unit of analysis, I also have concerns with respect to the sample. The sample is composed of open source software organizations, and the research question is appropriately scoped to state that only the performance of these 15 organizations is considered. However, it is unclear how organizations were selected for the sample. The selection seems non-random given that there are five of each size, with no short-lived or recently-formed organizations represented; i.e. there is bias due to both left and right censoring. To what extent do these organizations represent the population from which they were drawn? If this is a highly non-representative sample, then given the small number of organizations, it seems best framed as a set of quantitative case studies, not a study of FOSS organizations as a whole, and not of the FOSS movement as a whole. The analysis and all associated claims would then need to be revised to match. To my mind it is a powerful set of case studies. However, the data would need to be presented without being aggregated and analytic comparisons would need to be carefully done, perhaps by standardizing before aggregation and comparison, e.g. in standard deviation units.

3. Measures

The division of organizations into small, medium, and large seems arbitrary and the cut points may be driving the conclusion. The validity of any comparative work hinges on whether the basis of comparison is viable. Aggregation obscures differences between units, so it needs a fair amount of justification and alternatives explored and discarded in a robust way. At face value, it seems like there would be little benefit to comparing the work of these organizations by first aggregating them by number of projects. Debian is in "medium" but its work is unusual as it is primarily an integrator of myriad other projects, although it must sometimes develop tools to facilitate that work. Wikimedia is in "large" but is likely primarily a single project focused on content collection and display, although perhaps it has a bunch of little supportive tools, or perhaps the single project is represented as multiple projects in the dataset as an strategy for organizing the repository. Mozilla by contrast is "medium," but unlike Debian and Wikimedia it is focused on direct development of the sort that the paper is most concerned about, where a lines of code/commits analysis seems most apt; it's rather like Apache in this way. GNOME by contrast is more like a project than an organization, although it is in "small" alongside Gentoo, which is more like Debian despite being in "small". Given that a core -- and intriguing -- result of this paper relies on a distinction between sizes, it is important that the categorization scheme reflect something very meaningful about these organizations.

4. The methods employed for analysis

The paper uses a series of metrics typically used at the project level -- comments per line of code and lines per commit. Aggregating these metrics at a per-organization level gives a sense of how active the organization is at a given time, but when used comparatively through time it

also seems likely to reflect which projects each organization is facilitating, and this number is not stable over time.

In order to conclude that large organizations are declining at a greater rate than smaller organizations, the analysis would need to standardize within the organization and then compare it to other organizations using size as an important control. Given that project count changes over time as projects are founded, joined, divided, and end-of-lifed, any analysis should take this variation into account.

The paper cross-tabulates descriptive statistics, but no analysis is conducted to indicate whether a given size of organization is experiencing a statistically significant change over time. The concern for statistical significance was raised in prior reviews; I suggest that rather than working on the language to be even more thoroughly descriptive, a different analytical approach be taken. Analyzing multi-level time series data of this kind is a substantial undertaking, but I am concerned that claiming a result with respect to size and time will require more rigorous methods than what has been tackled here. For an example of how an analysis in a different peer production context has overcome similar issues to gain insight into a "rise and decline" pattern, I highly recommend the study in reference [1].

5. The analysis versus discussion points and conclusions

Success of Peer Production
The discussion opens by considering whether FOSS has served to "alter the fabric of late capitalist society". It is not clear from the data presented that any of the organizations analyzed took this as an ambition, or that a slowing in growth among a selection of organizations provides evidence on this topic. It may be argued instead that the explosive growth of the internet and the technology sector, enabled by FOSS, has indeed been transformative. There are careful distinctions to be made between the various organizational forms that FOSS projects may reside within and the paradigm, movement, etc. In any case the evidence developed in this work does not include relative market share measurements, relationships between projects and organizations, firms and their relationship to FOSS, or any changes within FOSS organizations.

FOSS or peer production as an approach may indeed be in a state of stability or decline, but the sample as constructed cannot tell us about this question. Evaluation of the paradigm (besides being a further unit of analysis) is different from analyzing the growth-stability/decline pattern typical of any organization or project -- just as growth metrics for 15 individual firms would be an unlikely proxy for the success of capitalism overall.

Given that all the organizations included in the analysis are at least 14 years old, it may be that newer organizations have taken their place, or that newer projects are drawing contributors while older and more established projects are in a stable or maintenance phase. By including only older organizations and aggregating projects, these distinctions are not visible. A similar issue arises for projects not in organizations, as mentioned in the limitations section -- however,

I think this goes beyond a limitation section mention; I think it needs to temper the claims to be made from the analysis.

Ultimately, a data-driven analysis of the success of the FOSS paradigm would be a very welcome contribution, but the research question and design of this work as conducted are not oriented to this.

Co-Option by Firms and FOSS Organizations Losing Salience
The discussion next explores concerns about co-option by firms and the work of FOSS organizations. This is an important line of inquiry, however here also the sample and analysis seem disconnected from the argument. No data or analysis is presented with respect to firms, including the relationship between firms and open source organizations, or any evidence of a timing in transformations -- the analysis does not present these dynamics, or any evidence of firms catching up versus FOSS organizations losing momentum. If suitable data with respect to firms was developed, this argument might then be made descriptively or be supported in a more rigorous way with the aid of vector autoregression (VAR) models.

The paper argues that some functions of organizations may be replaced by tools and platforms and that the need for the organizations is declining -- but no evidence is presented for this claim, which at the end of the discussion is called a 'nail in the coffin'. It seems reasonable that organizations might make use of tools and platforms in ways that make them more effective and efficient or the overall ecosystem more resilient. I'm not sure what platform is "the nail" in this discussion; the claim needs evidence.

The article goes on to argue that to the extent organizations are in decline, this may represent a lack of interest on the part of the public especially with respect to more ideological considerations. However, the article does not present evidence with respect to the public or ideology, but rather discusses how much work is done by participants inside projects inside 15 organizations.

The article offers a series of examples of services that are corporate or semi-corporate and rely on public content contributions, and disconnections between FOSS ideology and day-to-day concerns of tech users. These are interesting and important topics, but this line of argument seems unrelated to the article's analysis of organizational aggregate code productivity; it seems unlikely that a task like posting a review of a product is in competition with a task like coding a new feature for Firefox. One additional perspective that may be of interest with respect to how firms and open source relate to one another is reference [2].

6. Conclusions drawn

The title of the conclusion -- "a new morphology of FOSS social structures" is an interesting idea, but it does not seem apt as a section title -- the conclusion does not present a morphology or social structures. The abstract and conclusion summarize the following claims of the piece:

- that the relationship between peer production and commercial production is maturing -- however the paper does not develop evidence about relationships between these modes of production, or measure maturity, or examine any entities beyond the 15 FOSS organizations
- the role of large organizations as intermediaries is declining -- however the nature of this intermediation is not described (between whom and what do the organizations intermediate?), nor is evidence of a decline in intermediation developed
- the "likelihood of new big formal organizations" managing "emerging" projects is "systematically declining" -- however this assumes that organizations consider emerging projects to be within their scope; organizations in FOSS have often built up around the projects they manage and presumably emerging projects would likewise form their own organizations. The study does not examine newer organizations, or the likelihood of organizations forming; there is no data about projects that emerge outside organizations or about the conditions under which a project founded without an organization would then form or join an organization. The absence of projects outside organizations is listed as a limitation of the study so it should not be in a conclusion the study goes on to draw.
- open organizing is "a pipe dream"; this seems hyperbolic -- a pipe dream is one that has never come to pass, and the paper itself develops substantial evidence that open organizing has been instrumental in a range of high-impact projects (e.g. the Internet, Linux, Wikipedia). If an idiomatic expression is called for here, perhaps a more apt expression would be "a flash in the pan". Even that much deserves a degree of doubt; ultimately the paper does not conduct an evaluation of the organizing model, the relationship between firms and FOSS, the workings of co-option to disentangle who is co-opting whom, etc.

References

[1] Nathan TeBlunthuis, Aaron Shaw, and Benjamin Mako Hill. 2018. Revisiting "The Rise and Decline" in a Population of Peer Production Projects. In Proceedings of the 2018 CHI Conference on Human Factors in Computing Systems (CHI '18). Association for Computing Machinery, New York, NY, USA, Paper 355, 1–7. DOI:https://doi.org/10.1145/3173574.3173929

[2] Matt Germonprez, Jonathan Lipps, and Sean Goggins. 2019. The rising tide: Open source's steady transformation, First Monday, 24, 8, DOI: https://doi.org/10.5210/fm.v24i8.9297
