# Supplementary material for: Free and Open Source Software organizations: A large-scale analysis of code, comments, and commits frequency
Source: PLoS One. 2021 Sep 23;16(9):e0257192. doi: 10.1371/journal.pone.0257192 (PMC8460050; doi:10.1371/journal.pone.0257192)
Supplement: Attachment — Submitted filename: REPLY TO REVIEWERS PLOS third revision.docx [file pone.0257192.s004.docx]

Review Comments to the Author

Please use the space provided to explain your answers to the questions above. You may also include additional comments for the author, including concerns about dual publication, research ethics, or publication ethics. (Please upload your review as an attachment if it exceeds 20,000 characters)

Reviewer #2: I would like to thank authors for the detailed response and for addressing my comments. In my opinion, the new version of the paper has improved with regard to the previous one. I therefore recommend its acceptance.

Reviewer #3: I found myself even more excited about this paper having reviewed the latest version and I appreciate the revision efforts and response to comments. This is valuable and informative work that I am eager to see succeed. However, some of my core concerns remain, particularly with respect to the claims made versus the evidence presented.

Content-related concerns:

1. Abstract. I reprise my earlier comments here -- the final claim of the abstract is unsustained and needs to be amended. With apologies for the format of the below:

A. "as...the role of large FOSS organizations serving as platforms diminishes" -- it's not clear what is meant by "platforms" here, or whether these organizations seek to serve as such, or whether this service is indeed declining. What's presented is a decline in productivity and growth by various metrics, not a decline in "role as platform".

*Admittedly, in some part of the text, we’ve referred to FOSS as a platform and in other as software. This could be confusing for readers and misunderstood. We’ve reviewed and removed the word “platform” from the abstract and text in general, in many cases replacing the word “platform” with a more specific reference to services like Github or BitBucket.*

B. "the likelihood of new formal organizations" -- the analysis presented in this paper explicitly does not address new organizations nor does it measure likelihoods.

*Right, thank you. Rephrased/removed.*

C. "managing the...projects emerging is systematically declining" -- the analysis does not address emerging projects; to claim a systematically declining likelihood, one would need evidence addressing the system rather than only its long-lived members (to capture 'systematically'), as well as measuring which organizations an emerging project goes on to join (to capture 'likelihood').

*Corrected, we only claim that the activities in certain types of FOSS Organizations are declining. Additionally, based on your comment, we’ve added the disclaimer that there might be a new emerging FOSS organization outside the analyzed data that we don’t know about.*

2. Evidence-Claims Link. The "organizations supplanted by platforms" argument is problematic and emblematic of the necessity to connect claims and evidence I cited in my previous review. The use of the term "platform" is unclear and the argument of platforms supplanting organizations is not sustained by the analysis presented.

*Agreed. Please see our response in 1A - we removed the unfortunate and imprecise phrasing about platforms.*

A. In the abstract, organizations are said to have a role to serve as platforms; 1A has the opener for my points on this. Organizations create and use tools like GitHub to manage their work, but Mozilla for example is not Git nor does it frame itself as a Git provider.

*Agreed, per previous response.*

B. The paper presents an argument that "platforms" are supplanting "organizations" (p. 16). This claim needs evidence. It may be the case that new project founders don't see a need to join an organization when a platform is sufficiently robust, but evidence for this is not presented because of the way the sample is constructed. Valid evidence here would perhaps look like joining rates of new projects through processes like the Apache Incubator versus similarly-successful projects staying independent. But in this case, there's still no supplanting per se because this is a false dichotomy: every project needs both an organization of some kind and an instance on github or similar, with sufficient value in terms of users and code already accumulated before they could hope to join any organization.

*Agreed, we’ve revised the text to make sure we do not make claims outside the boundaries of analyzed data. Side note - we don’t have measurable evidence, but as you’ve mentioned - it looks like Github and similar services are effectively decentralizing previously formally organized Open Source Software.*

3. Sample. The response to my comments with respect to the sample are very much appreciated; a few more of these details should make their way into the paper, given the effort that was expended in this regard. I don't think it's necessary to include the data source in the title---my concern here is more about substance: reporting what is known about the sample, how it is constructed and vetted and verified, its limitations, and so on.

*Thank you, we’ve returned to the original title as suggested.*

4. Collection of minor points:

A. The paper would benefit from a modest revision pass for typographical issues. I observed single-sentence paragraphs and some of the captions had typos. Y-axis labels on the figures would be useful.

Thank you for this note, we’ve tested on a few random readers and decided to regenerate the figures adding more X/Y Axis data and making the graphs simpler and more clear.

B. Figure 4 is difficult to make sense of; it may be possible to improve it using a faceted plot or simply a repeat of the style of Figure 3.

We’ve regenerated Figure 3 with new styling and more detailed labels.

C. I'm not sure it's necessary to publish the QQ plot; a direct visualization of the distribution or a measure of skewness would be reasonable.

Agreed, The QQ plot has been removed for clarity and the remaining figures of remuneration has been changed.

D. "technological platforms" (p. 17) seems like an overbroad scope; I think what's meant here is only source control/collaboration platforms like GitHub.

Corrected.
